# Mechanisms of species diversity in birdsong learning

**DOI:** 10.1371/journal.pbio.3000555

**Published:** 2019-12-02

**Authors:** Sarah Cushing Woolley, Jon Tatsuya Sakata

**Affiliations:** 1 Department of Biology, McGill University, Montreal, Québec, Canada; 2 Center for Research on Brain, Language, and Music, McGill University, Montreal, Québec, Canada

## Abstract

Vocal communication is critical for social interactions across a diversity of animals. A subset of those animals, including humans and songbirds, must learn how to produce their vocal communication signals. In this issue of *PLOS Biology*, Wang and colleagues use genome-wide investigations of gene expression in species hybrids to uncover transcriptional networks that could influence species differences in song learning and production. We provide an overview of birdsong learning and discuss how the study by Wang and colleagues advances our understanding of mechanisms of song learning and evolution.

## Abstract 2

This Primer explores vocal learning in songbirds, focusing on the use of cross-fostering and species hybrids methods employed in a recent study to uncover transcriptional networks important for between-species differences in song learning and production.

A tremendous diversity of sounds for communication are used across animal species, and often these signals are sufficiently distinct that they can be used for species recognition. Whereas the production of communication signals does not require specific experiences or learning in many species, some species, such as humans, must learn their communication signals (reviewed in [1,2]). Researchers have long been fascinated with the remarkable plasticity involved in vocal learning and with the degree to which vocal learning shapes cultural evolution. However, less attention has been paid to the processes that bias and constrain the ability of young vocal learners to mimic the sounds and patterns of others and that maintain species differences in communication signals. For example, human infants are masters at acquiring language, and this predisposition for language acquisition hints that genes sculpt what young individuals can and will learn [3].

Songbirds are one the few vertebrate taxa that, like humans, learn their vocalizations. Broadly speaking, vocal learning in songbirds (as well as in other species) involves 2 learning processes—sensory learning and sensorimotor learning. In a typical process of vocal learning, young songbirds first memorize the song of an adult bird (“tutor”) during a developmental period of sensory learning. Then, songbirds undergo a period of sensorimotor learning (vocal motor practice) in which they refine their initially noisy vocalizations to match the memorized song. Songbirds continue to hone their vocalizations throughout development, and by the time they are adults, they can produce a song that bears great resemblance to the memorized song. Being able to hear others and oneself is critical in this process: individuals that are raised in isolation from song or prevented from hearing themselves sing during development fail to produce mature, species-typical vocalizations as adults (reviewed in [2,4]). Moreover, decades of research have revealed that sensory and sensorimotor aspects of vocal learning are controlled by specialized neural circuits, including primary and secondary auditory cortical regions; vocal motor areas similar to mammalian motor and premotor cortices; and a cortical-basal ganglia-thalamic loop (Fig 1) [2]. Revealing the processes by which these neural circuits encode and use the memorized song(s) to shape vocal imitation remains a central focus in the neuroethology of song learning [5].

**Fig 1:**
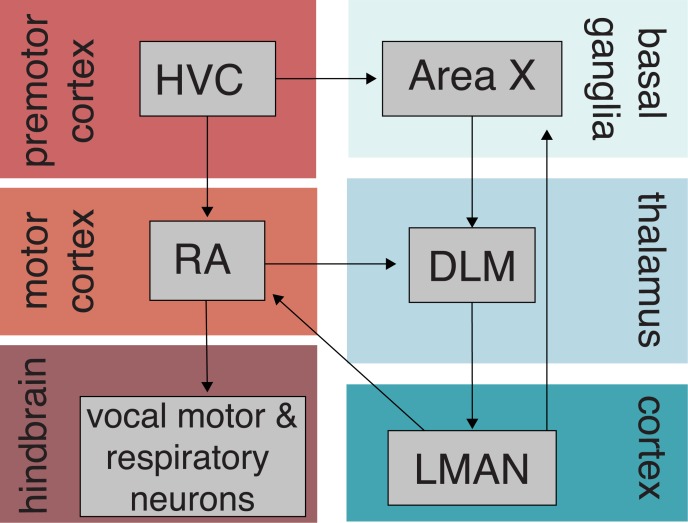
Neural circuits for birdsong learning and production. The canonical circuitry underlying song learning and control (“song system”) is parsed into the vocal motor pathway (red and orange boxes), which includes HVC (used as a proper name), RA, and hindbrain areas that contain vocal motor and respiratory neurons, and the anterior forebrain pathway (blue boxes), which includes the vocal basal ganglia nucleus Area X, DLM, and LMAN. HVC (which is thought to be analogous to the premotor cortex in mammals) and RA (which is thought to be analogous to parts of the primary motor cortex [22,30]) are critical for adult song production and implicated in species variation in song. DLM, medial portion of the dorsolateral thalamic nucleus; LMAN, lateral magnocellular nucleus of the anterior nidopallium; RA, robust nucleus of the arcopallium.

However, from the earliest observations of vocal learning in songbirds, an equally important aim has been to reveal the types of acoustic structures and sequences that birds cannot or do not learn and the biological mechanisms underlying species variation in song [6,7]. There are over 4,000 species of songbirds, each recognizable to members of their own species by the acoustic features and patterns of their songs. Just as in humans, songbirds preferentially learn the communication signals of their own species [1]. From an evolutionary standpoint, song is hypothesized to serve as a prezygotic isolation mechanism, a way for individuals to recognize other members of their species and avoid wasting time and energy in mating with members of a different species [8,9]. For song to function effectively in reproductive isolation, species must avoid learning the songs of heterospecifics that share the same habitat and, instead, preferentially learn the songs of conspecifics. This bias has long been assumed to reflect genetic influences on song learning.

Two experimental approaches have historically been used to reveal genetic biases in and constraints on song learning. The first is to cross-foster young birds of one species to parents of a different species that sings a distinct song. Cross-fostering elucidates both the potential plasticity (based on what birds are able to learn from heterospecific foster parents) and the constraints (based on what species-typical features birds retain from their own species) of song learning. When cross-fostered to and tutored by a different species, a number of songbirds can learn to accurately reproduce the acoustic elements of heterospecific songs [10–16]. However, even when exposed only to the songs of a heterospecific foster species, many songbirds retain hints of their species-typical structure when they imitate their foster parent’s song (Fig 2A). For example, chaffinches that are cross-fostered to canary parents are able to learn to produce the acoustic elements within canary song; however, they perform these elements with chaffinch-typical phrases or timing [16]. These cross-fostering experiments underscore that experience alone is not sufficient for songbirds to fully mimic the song of a different species and, moreover, that genes could shape the trajectory and nature of vocal learning.

**Fig 2:**
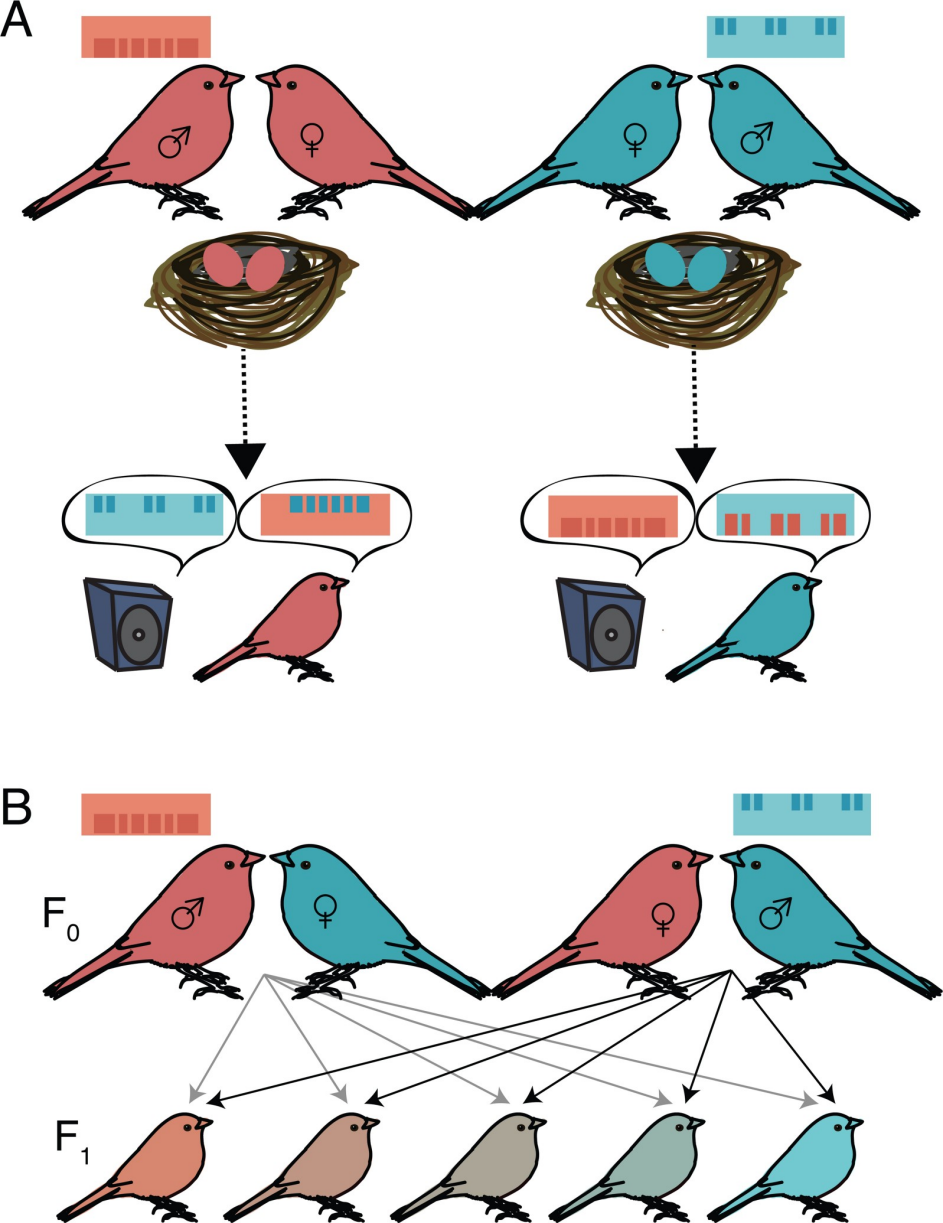
Cross-fostering and species hybrids lend insight into genetic constraints on and mechanisms of learning. (A) Cross-fostering young songbirds with a different songbird species can provide information on both the degree of plasticity in learning (based on how much song juvenile birds copy from their heterospecific tutor) and constraints on learning (based on how much of their species-typical song juvenile birds retain). In this example, which illustrates the experiment by Wang and colleagues, birds are removed from the nest and then tutored with the song of a different species played out from a speaker. In the illustrated song, spectrograms and colored bars represent the song’s spectral information, and the background highlights temporal information. A number of studies in songbirds demonstrate that cross-fostered birds learn the spectral properties of heterospecific songs (illustrated by the color and height of the bars) but retain several temporal properties of their own species (illustrated by the background color and spacing between elements). (B) Studies of song learning in species hybrids also indicate genetic contributions to learning. First filial (F1) hybrids (generated using both directions of hybridizations) vary in their allele-specific expression ratio toward each parent species. This variation may lead to differences in behavior that can be correlated with genetic markers and gene expression.

A complementary approach to reveal genetic contributions to song learning is to generate hybrids between 2 species that produce acoustically distinct songs (Fig 2B) [15,17,18]. The earliest studies of hybrids focused on behaviors with a minimal experiential component, whereas more recent work has provided detailed examinations of genetic and genomic contributions to learned behaviors, including the sensory and sensorimotor learning of birdsong. For example, hybridizations and backcrosses between border and roller canaries, 2 artificially selected strains of canary that differ in their tendency to sing high-pitched (border) or low-pitched (roller) song phrases, have revealed a contribution of autosomal and sex-linked genes to species variation in song phrases [18].

Together with candidate gene approaches [19–21], cross-fostering and species hybrid approaches have helped reveal the existence of genetic influences on song learning. More recently, advances in both genomic and computational tools [22–24] have made it possible to assess the degree to which species differences in gene regulatory networks underlie species variation in vocal communication. Differences in gene expression, thought to contribute to phenotypic differences within and between species, can result from cis- and/or trans-acting regulatory differences. Changes in cis-regulatory elements include changes to promoter and gene sequences and messenger RNA stability, whereas trans effects reflect changes to diffusible factors including transcription factors, microRNAs, and chromatin regulators [24]. In songbirds, species differences in transcriptional regulation within brain areas that are critical for song learning and production (Fig 1) may contribute to the bias to learn species-typical songs.

To uncover the degree to which species divergences in regulatory networks could underlie species differences in song, Wang and colleagues (this issue) [25] integrated cross-fostering, species hybridizations, and genome-wide transcriptional analysis of 2 related songbirds—zebra finches and owl finches. The authors first cross-fostered juvenile zebra finches and owl finches and demonstrated a strong signature of genetic constraints on song learning (Fig 2A). Then, they hybridized zebra and owl finches and performed a genome-wide transcriptional analysis of 2 key song nuclei (HVC and robust nucleus of the arcopallium [RA]; see Figs 1 and 2B) in F1 hybrids to elucidate divergences in transcriptional regulatory networks between the 2 species. They found that divergence in transcriptional regulation accounted for approximately 10% of the variation in transcribed genes. Moreover, in contrast to many studies that highlight cis-regulatory changes as central to the evolution of gene expression (reviewed in [26]), Wang and colleagues discovered that trans-regulatory changes were more prevalent than cis changes. Interestingly, these trans-regulatory changes in brain areas important for song production were associated with genes involved in synapse formation and transmission.

One gene in particular, brain-derived neurotrophic factor (BDNF), was identified as an upstream mediator of a substantial number of trans-regulated genes in the vocal motor pathway. Moreover, there were species differences in both amino acid substitutions and BDNF expression levels, and Wang and colleagues examined the degree to which these species differences related to species differences in songs. They found that individual differences in acoustic features of song of F1 hybrids were more correlated with individual differences in the level of BDNF expression in HVC and RA than with the allele-specific expression (ASE) ratio, which associates with differences in amino acid substitution of the parental species. In addition, they found that manipulating BDNF in RA in the adult zebra finch by continuous, local administration of a BDNF agonist led to changes to syllable structure and sequencing in adult zebra finch song (see also [27,28]) and affected the expression of over 500 downstream genes, including a subset of the genes putatively regulated by BDNF.

Taken as a whole, the study by Wang and colleagues represents significant progress in understanding the genetic and genomic underpinnings of species differences in vocal motor learning and production. Moreover, their data highlight a number of exciting directions to extend their findings and further elucidate the genetic bases of song learning. For example, future studies should focus on juveniles that are undergoing vocal learning to further examine both the identified factors from this study as well as search for additional transcriptional variation. Understanding how development and experience shape gene regulation and expression will provide deeper insights into mechanisms of song learning and evolution. In addition, because genes influence sensory acquisition and sensorimotor development [10,12,13,15,18,29], future work revealing how genes and regulatory networks for sensory and sensorimotor learning vary between species will be essential for understanding how species differences in song arise.

Finally, the study by Wang and colleagues also highlights an inherent challenge in the hunt for the genetic bases of species differences in behavior. Elucidating how complex gene regulatory networks evolve and how their evolution results in phenotypic change and speciation is a monumental task, especially when it involves phenotypes as complex, multidimensional, and dynamic as vocal learning and production. As our toolbox grows, and as studies move away from focusing on a handful of traditional candidate genes toward investigating gene regulatory networks, we will gain greater resolution into the multifaceted relationships between gene expression and species-specific behaviors, even for learned behaviors such as birdsong.

## Abbreviations

ASE: allele-specific expression
BDNF: brain-derived neurotrophic factor
DLM: medial portion of the dorsolateral thalamic nucleus
LMAN: lateral magnocellular nucleus of the anterior nidopallium
RA: robust nucleus of the arcopallium
